# Supplementary material for: Content-rich biological network constructed by mining PubMed abstracts
Source: BMC Bioinformatics. 2004 Oct 8;5:147. doi: 10.1186/1471-2105-5-147 (PMC528731; doi:10.1186/1471-2105-5-147)
Supplement: Additional File 5 — The original Chilibot query results of the term "long-term potentiation (LTP)" and 22 other terms, limiting the latest references analyzed to the years 1990, 1995, 2000, and 2004. [file 1471-2105-5-147-S5.bz2 › chilibotAdditionalFile5/ltp2000/html/KV4.2_CAMKII.html]

 


 **KV4.2** and **CAMKII** 
  
Found 2 abstracts in PubMed,  **2 abstracts were retrieved and analyzed**.  


---

 Search Google  |
 PDF files only 
|  EDU domain only 

---

**Interactive relationship** (e.g. stimulation, inhibition, etc)

**Parallel relationship** (e.g. studied together, co-existance, homology, etc.)

- Phosphorylation consensus sites for various kinases exist within the sequence of the potassium channel subunit  **Kv4.2** , including consensus sites for extracellular signal regulated kinase mitogen activated protein kinase ERK MAPK, protein kinase A PKA, protein kinase C PKC, and calcium calmodulin dependent kinase II  **CaMKII** , and kinase assays have shown that particular amino acids of the consensus sites are bonafide phosphorylation sites in vitro.  Ref: 11040264 Learn MemLearn Mem, 1996
